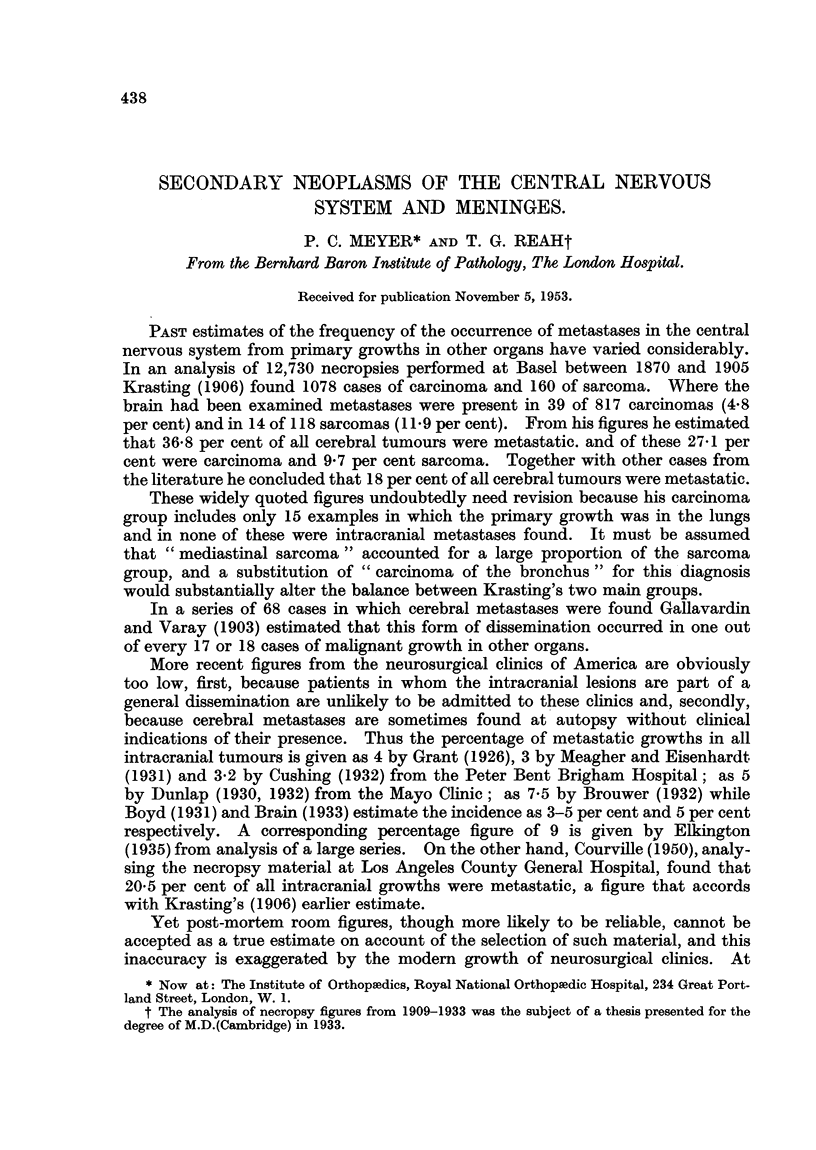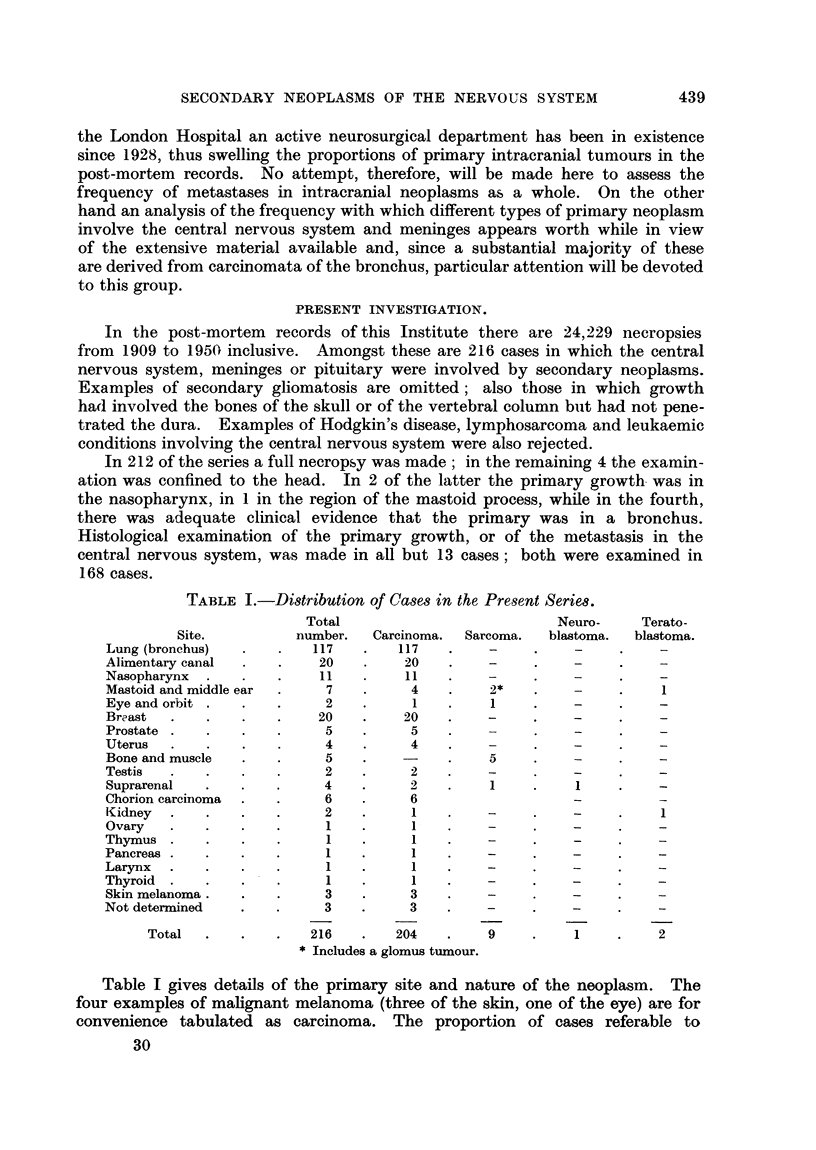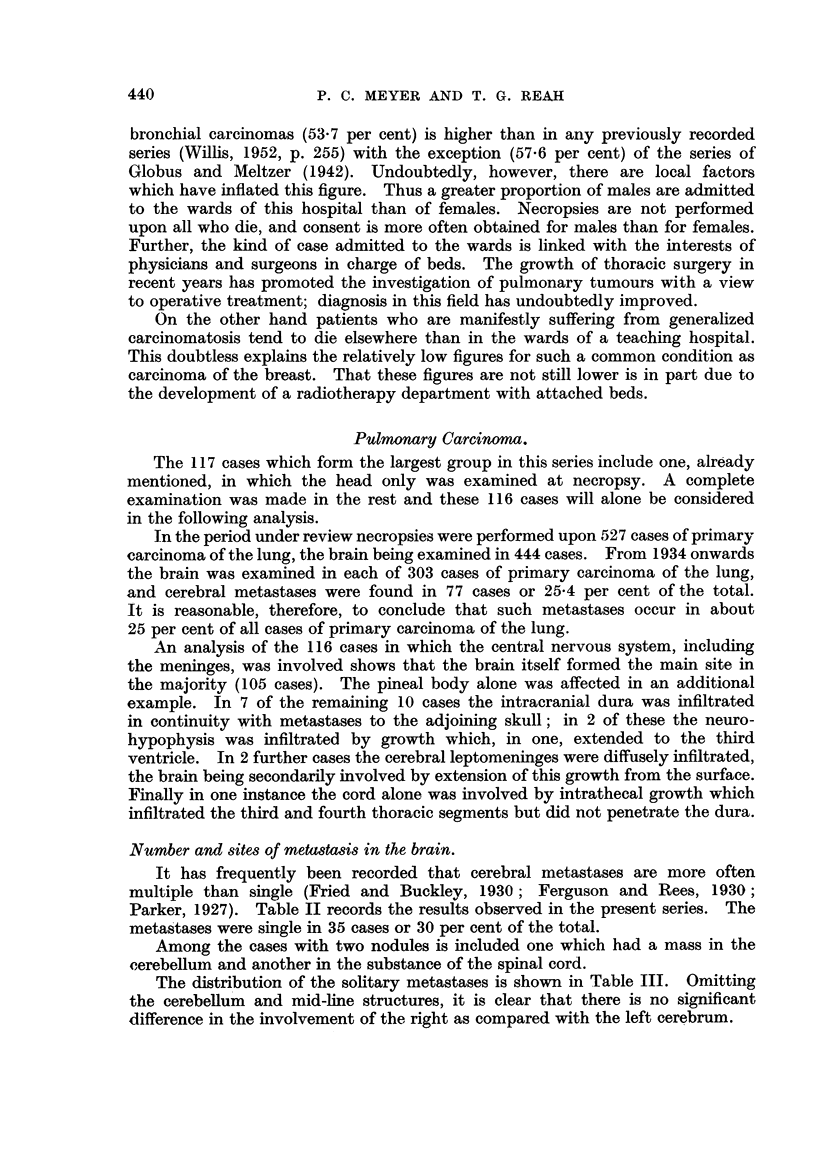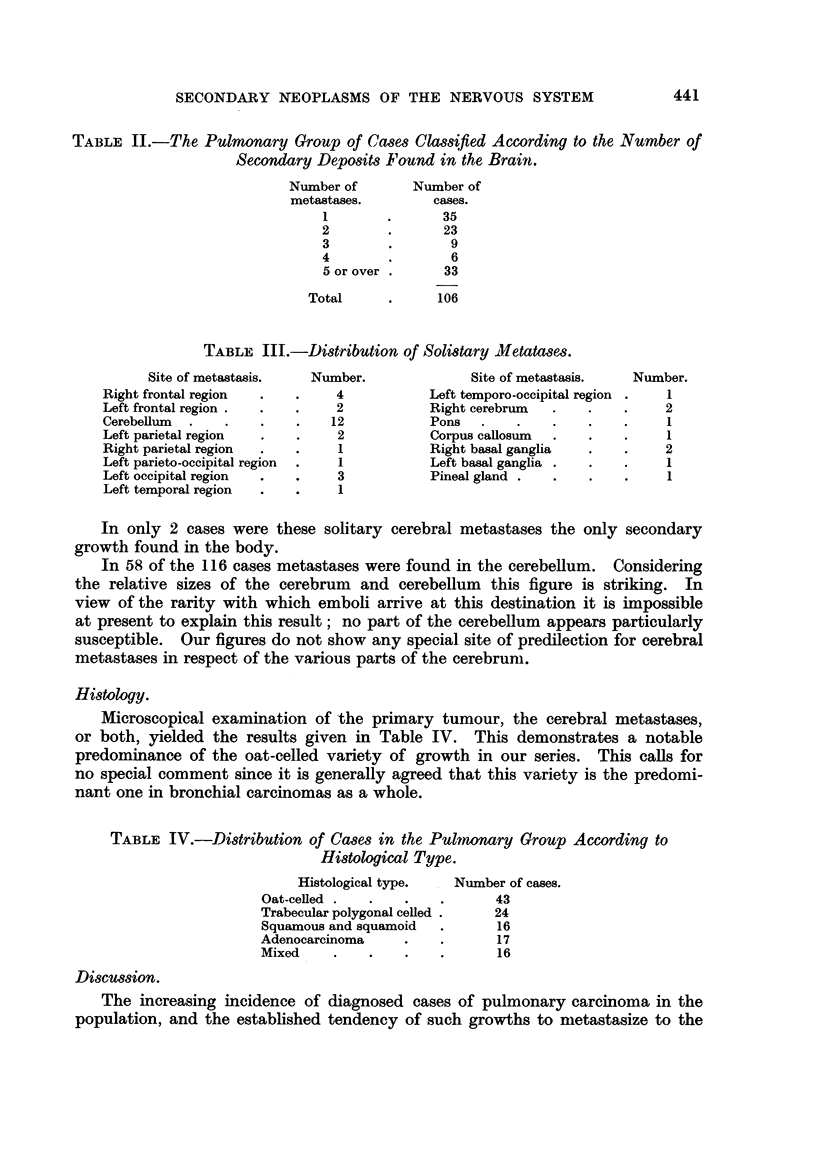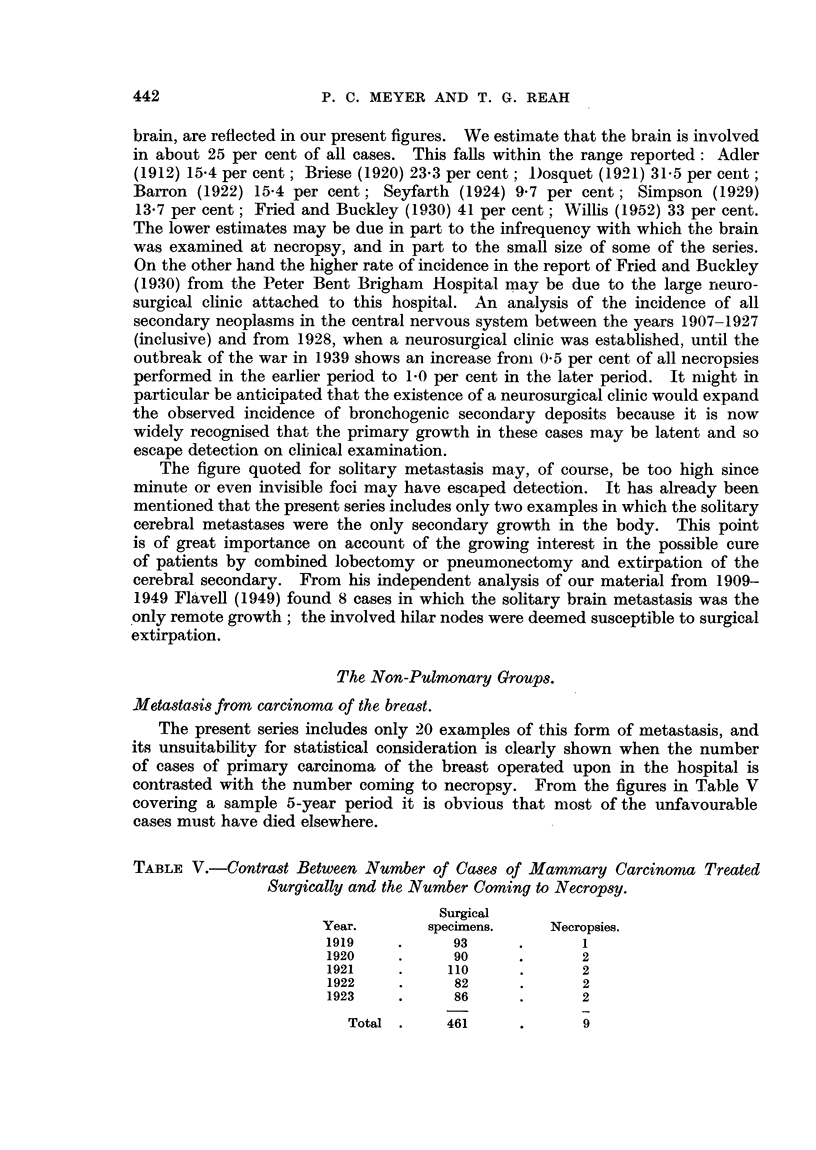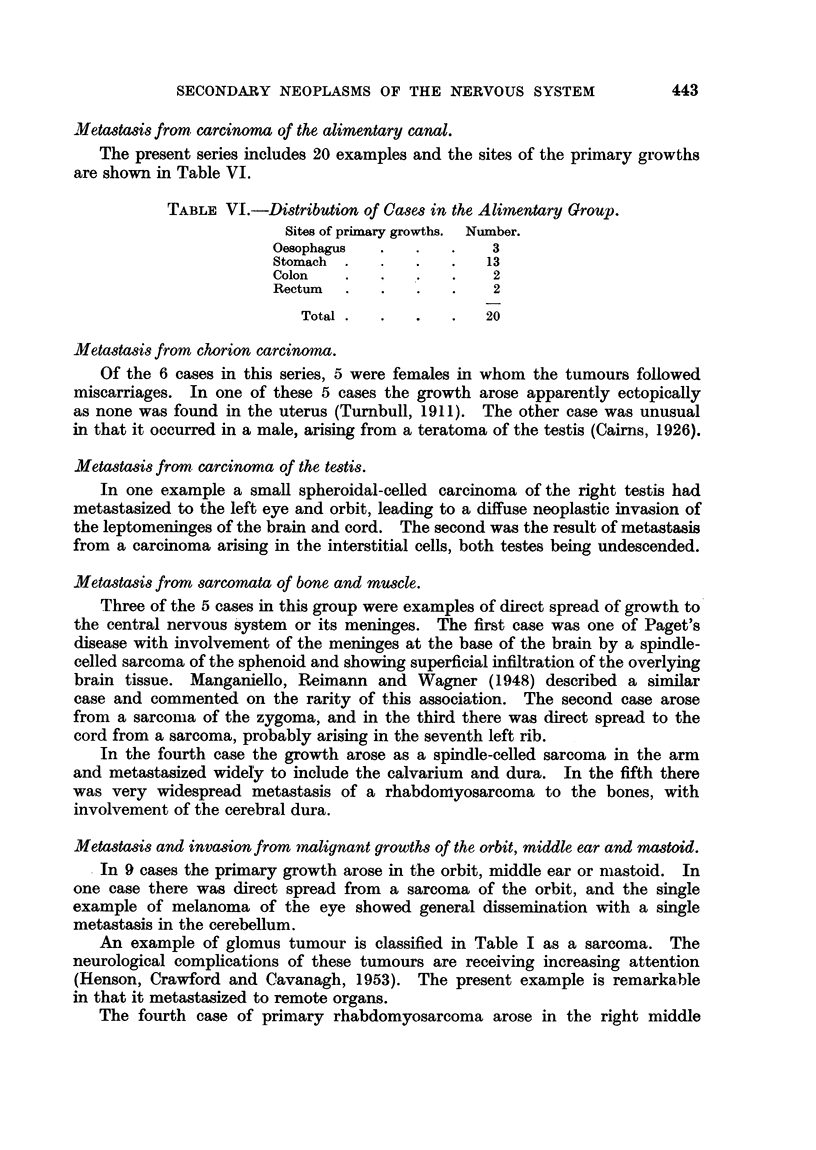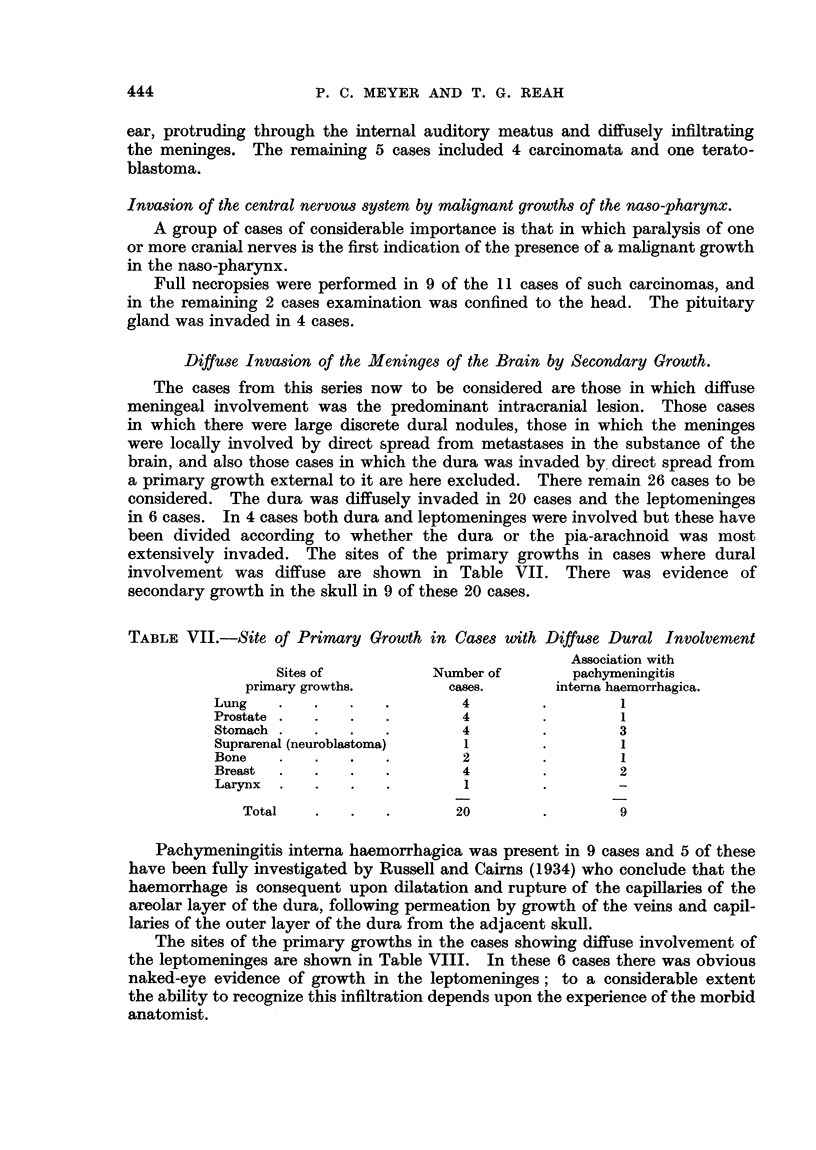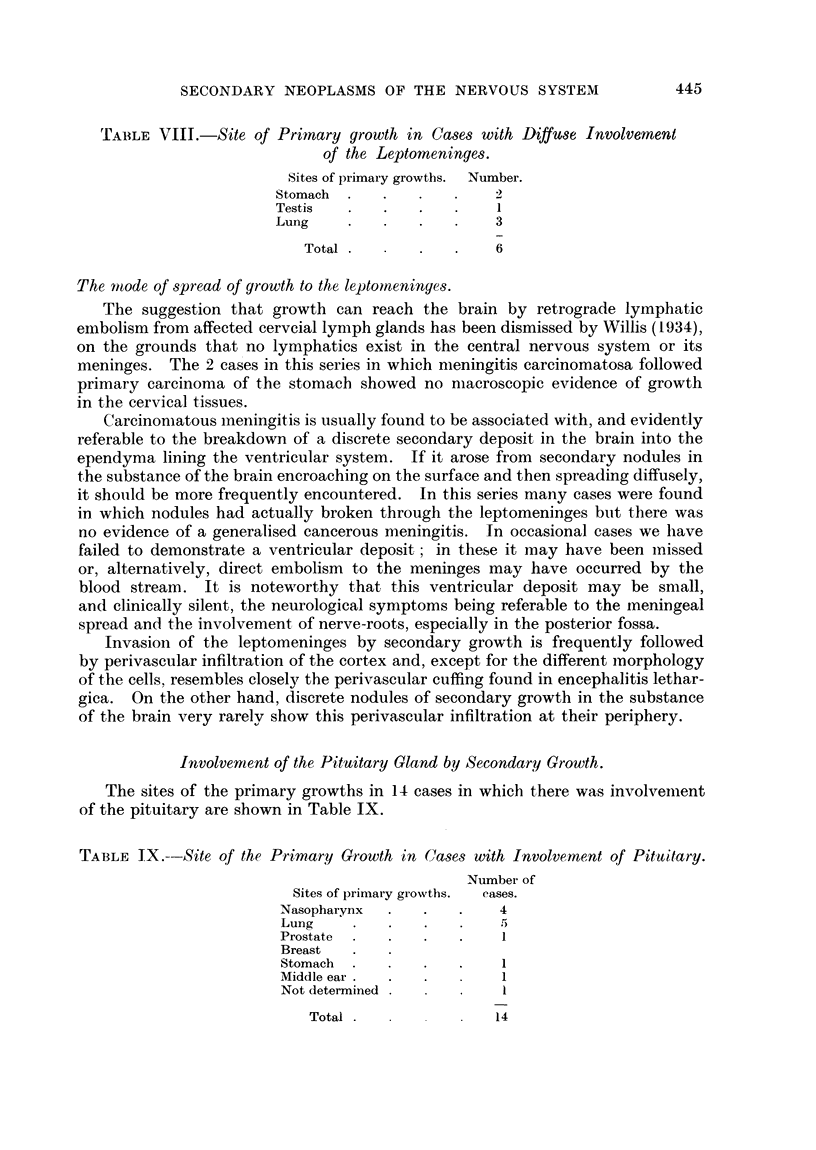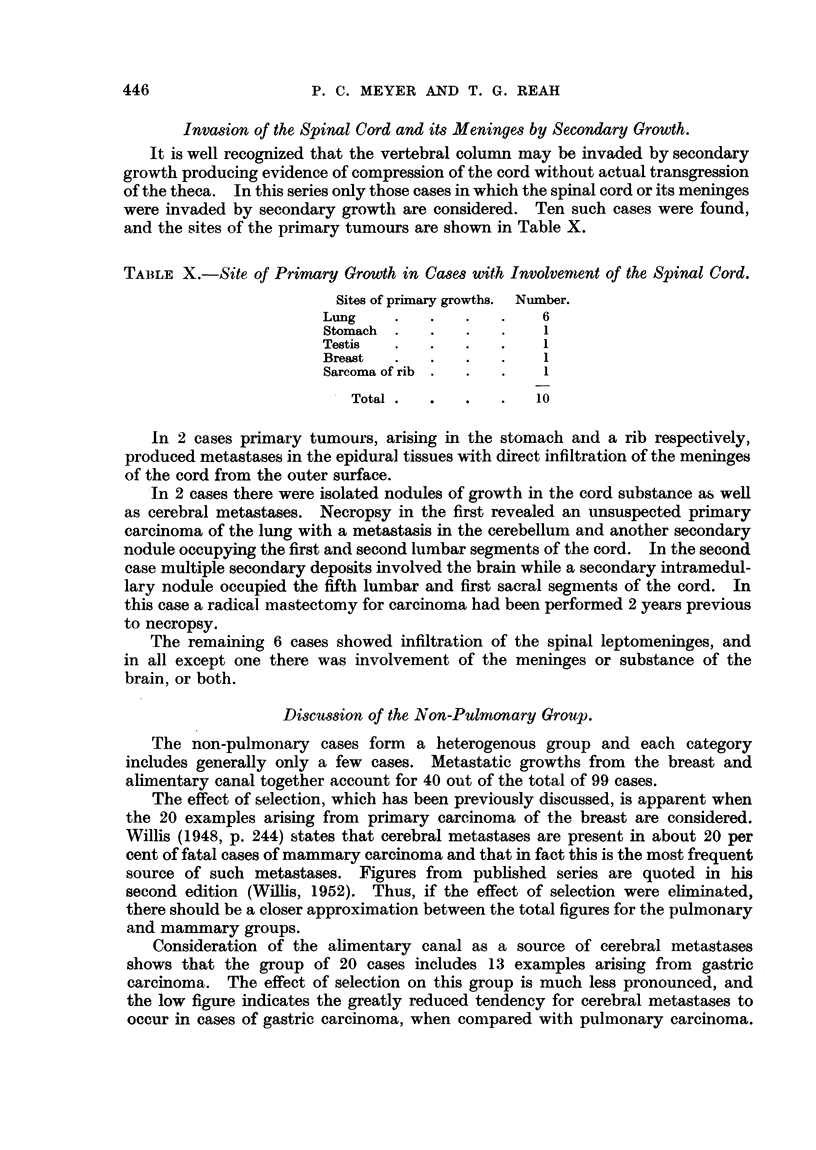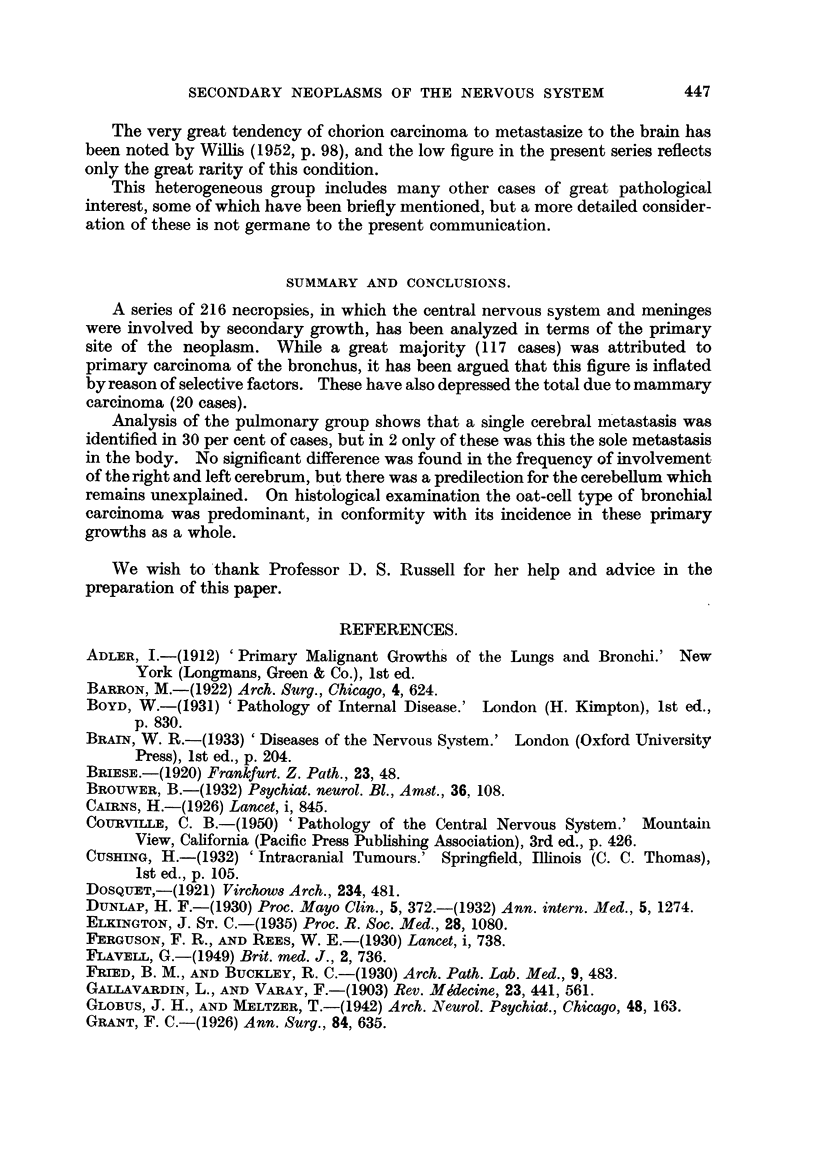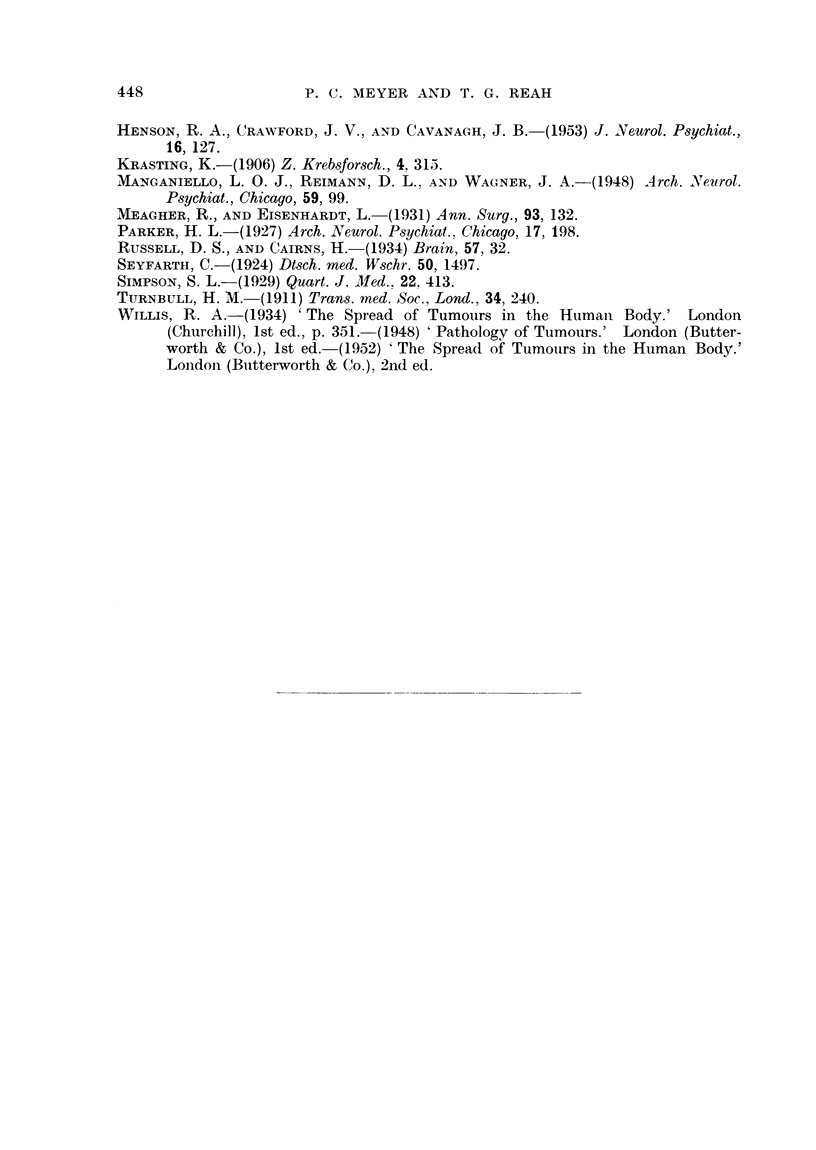# Secondary Neoplasms of the Central Nervous System and Meninges

**DOI:** 10.1038/bjc.1953.44

**Published:** 1953-12

**Authors:** P. C. Meyer, T. G. Reah


### SECONDARY NEOPLASMS OF THE CENTRAL NERVOUS SYSTEM AND MENINGES.

P. C. MEYER* AND T. G. REAH†

*From the Bernhard Baron Institute of Pathology, The London Hospital.*

Received for publication November 5, 1953.

PAST estimates of the frequency of the occurrence of metastases in the central nervous system from primary growths in other organs have varied considerably. In an analysis of 12,730 necropsies performed at Basel between 1870 and 1905 Krasting (1906) found 1078 cases of carcinoma and 160 of sarcoma. Where the brain had been examined metastases were present in 39 of 817 carcinomas (4·8 per cent) and in 14 of 118 sarcomas (11·9 per cent). From his figures he estimated that 36·8 per cent of all cerebral tumours were metastatic. and of these 27·1 per cent were carcinoma and 9·7 per cent sarcoma. Together with other cases from the literature he concluded that 18 per cent of all cerebral tumours were metastatic.

These widely quoted figures undoubtedly need revision because his carcinoma group includes only 15 examples in which the primary growth was in the lungs and in none of these were intracranial metastases found. It must be assumed that " mediastinal sarcoma " accounted for a large proportion of the sarcoma group, and a substitution of " carcinoma of the bronchus " for this diagnosis would substantially alter the balance between Krasting's two main groups.

In a series of 68 cases in which cerebral metastases were found Gallavardin and Varay (1903) estimated that this form of dissemination occurred in one out of every 17 or 18 cases of malignant growth in other organs.

More recent figures from the neurosurgical clinics of America are obviously too low, first, because patients in whom the intracranial lesions are part of a general dissemination are unlikely to be admitted to these clinics and, secondly, because cerebral metastases are sometimes found at autopsy without clinical indications of their presence. Thus the percentage of metastatic growths in all intracranial tumours is given as 4 by Grant (1926), 3 by Meagher and Eisenhardt (1931) and 3·2 by Cushing (1932) from the Peter Bent Brigham Hospital; as 5 by Dunlap (1930, 1932) from the Mayo Clinic; as 7·5 by Brouwer (1932) while Boyd (1931) and Brain (1933) estimate the incidence as 3–5 per cent and 5 per cent respectively. A corresponding percentage figure of 9 is given by Elkington (1935) from analysis of a large series. On the other hand, Courville (1950), analysing the necropsy material at Los Angeles County General Hospital, found that 20·5 per cent of all intracranial growths were metastatic, a figure that accords with Krasting's (1906) earlier estimate.

Yet post-mortem room figures, though more likely to be reliable, cannot be accepted as a true estimate on account of the selection of such material, and this inaccuracy is exaggerated by the modern growth of neurosurgical clinics. At

* Now at: The Institute of Orthopædics, Royal National Orthopædic Hospital, 234 Great Portland Street, London, W. 1.

† The analysis of necropsy figures from 1909–1933 was the subject of a thesis presented for the degree of M.D.(Cambridge) in 1933.

the London Hospital an active neurosurgical department has been in existence since 1928, thus swelling the proportions of primary intracranial tumours in the post-mortem records. No attempt, therefore, will be made here to assess the frequency of metastases in intracranial neoplasms as a whole. On the other hand an analysis of the frequency with which different types of primary neoplasm involve the central nervous system and meninges appears worth while in view of the extensive material available and, since a substantial majority of these are derived from carcinomata of the bronchus, particular attention will be devoted to this group.

### PRESENT INVESTIGATION.

In the post-mortem records of this Institute there are 24,229 necropsies from 1909 to 1950 inclusive. Amongst these are 216 cases in which the central nervous system, meninges or pituitary were involved by secondary neoplasms. Examples of secondary gliomatosis are omitted ; also those in which growth had involved the bones of the skull or of the vertebral column but had not penetrated the dura. Examples of Hodgkin's disease, lymphosarcoma and leukaemic conditions involving the central nervous system were also rejected.

In 212 of the series a full necropsy was made ; in the remaining 4 the examination was confined to the head. In 2 of the latter the primary growth was in the nasopharynx, in 1 in the region of the mastoid process, while in the fourth, there was adequate clinical evidence that the primary was in a bronchus. Histological examination of the primary growth, or of the metastasis in the central nervous system, was made in all but 13 cases ; both were examined in 168 cases.

TABLE I.—*Distribution of Cases in the Present Series.*

| Site. | Total number. | Carcinoma. | Sarcoma. | Neuro-blastoma. | Terato-blastoma. |
|---|---|---|---|---|---|
| Lung (bronchus) | 117 | 117 | – | – | – |
| Alimentary canal | 20 | 20 | – | – | – |
| Nasopharynx | 11 | 11 | – | – | – |
| Mastoid and middle ear | 7 | 4 | 2* | – | 1 |
| Eye and orbit | 2 | 1 | 1 | – | – |
| Breast | 20 | 20 | – | – | – |
| Prostate | 5 | 5 | – | – | – |
| Uterus | 4 | 4 | – | – | – |
| Bone and muscle | 5 | — | 5 | – | – |
| Testis | 2 | 2 | – | – | – |
| Suprarenal | 4 | 2 | 1 | 1 | – |
| Chorion carcinoma | 6 | 6 | | – | – |
| Kidney | 2 | 1 | – | – | 1 |
| Ovary | 1 | 1 | – | – | – |
| Thymus | 1 | 1 | – | – | – |
| Pancreas | 1 | 1 | – | – | – |
| Larynx | 1 | 1 | – | – | – |
| Thyroid | 1 | 1 | – | – | – |
| Skin melanoma | 3 | 3 | – | – | – |
| Not determined | 3 | 3 | – | – | – |
| Total | 216 | 204 | 9 | 1 | 2 |

* Includes a glomus tumour.

Table I gives details of the primary site and nature of the neoplasm. The four examples of malignant melanoma (three of the skin, one of the eye) are for convenience tabulated as carcinoma. The proportion of cases referable to bronchial carcinomas (53·7 per cent) is higher than in any previously recorded series (Willis, 1952, p. 255) with the exception (57·6 per cent) of the series of Globus and Meltzer (1942). Undoubtedly, however, there are local factors which have inflated this figure. Thus a greater proportion of males are admitted to the wards of this hospital than of females. Necropsies are not performed upon all who die, and consent is more often obtained for males than for females. Further, the kind of case admitted to the wards is linked with the interests of physicians and surgeons in charge of beds. The growth of thoracic surgery in recent years has promoted the investigation of pulmonary tumours with a view to operative treatment; diagnosis in this field has undoubtedly improved.

On the other hand patients who are manifestly suffering from generalized carcinomatosis tend to die elsewhere than in the wards of a teaching hospital. This doubtless explains the relatively low figures for such a common condition as carcinoma of the breast. That these figures are not still lower is in part due to the development of a radiotherapy department with attached beds.

*Pulmonary Carcinoma.*

The 117 cases which form the largest group in this series include one, already mentioned, in which the head only was examined at necropsy. A complete examination was made in the rest and these 116 cases will alone be considered in the following analysis.

In the period under review necropsies were performed upon 527 cases of primary carcinoma of the lung, the brain being examined in 444 cases. From 1934 onwards the brain was examined in each of 303 cases of primary carcinoma of the lung, and cerebral metastases were found in 77 cases or 25·4 per cent of the total. It is reasonable, therefore, to conclude that such metastases occur in about 25 per cent of all cases of primary carcinoma of the lung.

An analysis of the 116 cases in which the central nervous system, including the meninges, was involved shows that the brain itself formed the main site in the majority (105 cases). The pineal body alone was affected in an additional example. In 7 of the remaining 10 cases the intracranial dura was infiltrated in continuity with metastases to the adjoining skull; in 2 of these the neurohypophysis was infiltrated by growth which, in one, extended to the third ventricle. In 2 further cases the cerebral leptomeninges were diffusely infiltrated, the brain being secondarily involved by extension of this growth from the surface. Finally in one instance the cord alone was involved by intrathecal growth which infiltrated the third and fourth thoracic segments but did not penetrate the dura.

*Number and sites of metastasis in the brain.*

It has frequently been recorded that cerebral metastases are more often multiple than single (Fried and Buckley, 1930; Ferguson and Rees, 1930; Parker, 1927). Table II records the results observed in the present series. The metastases were single in 35 cases or 30 per cent of the total.

Among the cases with two nodules is included one which had a mass in the cerebellum and another in the substance of the spinal cord.

The distribution of the solitary metastases is shown in Table III. Omitting the cerebellum and mid-line structures, it is clear that there is no significant difference in the involvement of the right as compared with the left cerebrum.

TABLE II.—*The Pulmonary Group of Cases Classified According to the Number of Secondary Deposits Found in the Brain.*

| Number of metastases. | Number of cases. |
|---|---|
| 1 | 35 |
| 2 | 23 |
| 3 | 9 |
| 4 | 6 |
| 5 or over | 33 |
| Total | 106 |

TABLE III.—*Distribution of Solistary Metatases.*

| Site of metastasis. | Number. | Site of metastasis. | Number. |
|---|---|---|---|
| Right frontal region | 4 | Left temporo-occipital region | 1 |
| Left frontal region | 2 | Right cerebrum | 2 |
| Cerebellum | 12 | Pons | 1 |
| Left parietal region | 2 | Corpus callosum | 1 |
| Right parietal region | 1 | Right basal ganglia | 2 |
| Left parieto-occipital region | 1 | Left basal ganglia | 1 |
| Left occipital region | 3 | Pineal gland | 1 |
| Left temporal region | 1 | | |

In only 2 cases were these solitary cerebral metastases the only secondary growth found in the body.

In 58 of the 116 cases metastases were found in the cerebellum. Considering the relative sizes of the cerebrum and cerebellum this figure is striking. In view of the rarity with which emboli arrive at this destination it is impossible at present to explain this result; no part of the cerebellum appears particularly susceptible. Our figures do not show any special site of predilection for cerebral metastases in respect of the various parts of the cerebrum.

*Histology.*

Microscopical examination of the primary tumour, the cerebral metastases, or both, yielded the results given in Table IV. This demonstrates a notable predominance of the oat-celled variety of growth in our series. This calls for no special comment since it is generally agreed that this variety is the predominant one in bronchial carcinomas as a whole.

TABLE IV.—*Distribution of Cases in the Pulmonary Group According to Histological Type.*

| Histological type. | Number of cases. |
|---|---|
| Oat-celled | 43 |
| Trabecular polygonal celled | 24 |
| Squamous and squamoid | 16 |
| Adenocarcinoma | 17 |
| Mixed | 16 |

*Discussion.*

The increasing incidence of diagnosed cases of pulmonary carcinoma in the population, and the established tendency of such growths to metastasize to the brain, are reflected in our present figures. We estimate that the brain is involved in about 25 per cent of all cases. This falls within the range reported: Adler (1912) 15·4 per cent; Briese (1920) 23·3 per cent; Dosquet (1921) 31·5 per cent; Barron (1922) 15·4 per cent; Seyfarth (1924) 9·7 per cent; Simpson (1929) 13·7 per cent; Fried and Buckley (1930) 41 per cent; Willis (1952) 33 per cent. The lower estimates may be due in part to the infrequency with which the brain was examined at necropsy, and in part to the small size of some of the series. On the other hand the higher rate of incidence in the report of Fried and Buckley (1930) from the Peter Bent Brigham Hospital may be due to the large neurosurgical clinic attached to this hospital. An analysis of the incidence of all secondary neoplasms in the central nervous system between the years 1907–1927 (inclusive) and from 1928, when a neurosurgical clinic was established, until the outbreak of the war in 1939 shows an increase from 0·5 per cent of all necropsies performed in the earlier period to 1·0 per cent in the later period. It might in particular be anticipated that the existence of a neurosurgical clinic would expand the observed incidence of bronchogenic secondary deposits because it is now widely recognised that the primary growth in these cases may be latent and so escape detection on clinical examination.

The figure quoted for solitary metastasis may, of course, be too high since minute or even invisible foci may have escaped detection. It has already been mentioned that the present series includes only two examples in which the solitary cerebral metastases were the only secondary growth in the body. This point is of great importance on account of the growing interest in the possible cure of patients by combined lobectomy or pneumonectomy and extirpation of the cerebral secondary. From his independent analysis of our material from 1909–1949 Flävell (1949) found 8 cases in which the solitary brain metastasis was the only remote growth; the involved hilar nodes were deemed susceptible to surgical extirpation.

### *The Non-Pulmonary Groups.*

*Metastasis from carcinoma of the breast.*

The present series includes only 20 examples of this form of metastasis, and its unsuitability for statistical consideration is clearly shown when the number of cases of primary carcinoma of the breast operated upon in the hospital is contrasted with the number coming to necropsy. From the figures in Table V covering a sample 5-year period it is obvious that most of the unfavourable cases must have died elsewhere.

TABLE V.—*Contrast Between Number of Cases of Mammary Carcinoma Treated Surgically and the Number Coming to Necropsy.*

| Year. | Surgical specimens. | Necropsies. |
|---|---|---|
| 1919 | 93 | 1 |
| 1920 | 90 | 2 |
| 1921 | 110 | 2 |
| 1922 | 82 | 2 |
| 1923 | 86 | 2 |
| Total | 461 | 9 |

*Metastasis from carcinoma of the alimentary canal.*

The present series includes 20 examples and the sites of the primary growths are shown in Table VI.

TABLE VI.—*Distribution of Cases in the Alimentary Group.*

| Sites of primary growths. | Number. |
|---|---|
| Oesophagus . . . | 3 |
| Stomach . . . . | 13 |
| Colon . . . . | 2 |
| Rectum . . . . | 2 |
| Total . . . . | 20 |

*Metastasis from chorion carcinoma.*

Of the 6 cases in this series, 5 were females in whom the tumours followed miscarriages. In one of these 5 cases the growth arose apparently ectopically as none was found in the uterus (Turnbull, 1911). The other case was unusual in that it occurred in a male, arising from a teratoma of the testis (Cairns, 1926).

*Metastasis from carcinoma of the testis.*

In one example a small spheroidal-celled carcinoma of the right testis had metastasized to the left eye and orbit, leading to a diffuse neoplastic invasion of the leptomeninges of the brain and cord. The second was the result of metastasis from a carcinoma arising in the interstitial cells, both testes being undescended.

*Metastasis from sarcomata of bone and muscle.*

Three of the 5 cases in this group were examples of direct spread of growth to the central nervous system or its meninges. The first case was one of Paget's disease with involvement of the meninges at the base of the brain by a spindle-celled sarcoma of the sphenoid and showing superficial infiltration of the overlying brain tissue. Manganiello, Reimann and Wagner (1948) described a similar case and commented on the rarity of this association. The second case arose from a sarcoma of the zygoma, and in the third there was direct spread to the cord from a sarcoma, probably arising in the seventh left rib.

In the fourth case the growth arose as a spindle-celled sarcoma in the arm and metastasized widely to include the calvarium and dura. In the fifth there was very widespread metastasis of a rhabdomyosarcoma to the bones, with involvement of the cerebral dura.

*Metastasis and invasion from malignant growths of the orbit, middle ear and mastoid.*

In 9 cases the primary growth arose in the orbit, middle ear or mastoid. In one case there was direct spread from a sarcoma of the orbit, and the single example of melanoma of the eye showed general dissemination with a single metastasis in the cerebellum.

An example of glomus tumour is classified in Table I as a sarcoma. The neurological complications of these tumours are receiving increasing attention (Henson, Crawford and Cavanagh, 1953). The present example is remarkable in that it metastasized to remote organs.

The fourth case of primary rhabdomyosarcoma arose in the right middle ear, protruding through the internal auditory meatus and diffusely infiltrating the meninges. The remaining 5 cases included 4 carcinomata and one teratoblastoma.

*Invasion of the central nervous system by malignant growths of the naso-pharynx.*

A group of cases of considerable importance is that in which paralysis of one or more cranial nerves is the first indication of the presence of a malignant growth in the naso-pharynx.

Full necropsies were performed in 9 of the 11 cases of such carcinomas, and in the remaining 2 cases examination was confined to the head. The pituitary gland was invaded in 4 cases.

### *Diffuse Invasion of the Meninges of the Brain by Secondary Growth.*

The cases from this series now to be considered are those in which diffuse meningeal involvement was the predominant intracranial lesion. Those cases in which there were large discrete dural nodules, those in which the meninges were locally involved by direct spread from metastases in the substance of the brain, and also those cases in which the dura was invaded by direct spread from a primary growth external to it are here excluded. There remain 26 cases to be considered. The dura was diffusely invaded in 20 cases and the leptomeninges in 6 cases. In 4 cases both dura and leptomeninges were involved but these have been divided according to whether the dura or the pia-arachnoid was most extensively invaded. The sites of the primary growths in cases where dural involvement was diffuse are shown in Table VII. There was evidence of secondary growth in the skull in 9 of these 20 cases.

TABLE VII.—*Site of Primary Growth in Cases with Diffuse Dural Involvement*

| Sites of primary growths. | Number of cases. | Association with pachymeningitis interna haemorrhagica. |
|---|---|---|
| Lung . . . . | 4 | 1 |
| Prostate . . . . | 4 | 1 |
| Stomach . . . . | 4 | 3 |
| Suprarenal (neuroblastoma) | 1 | 1 |
| Bone . . . . | 2 | 1 |
| Breast . . . . | 4 | 2 |
| Larynx . . . . | 1 | – |
| Total . . . | 20 | 9 |

Pachymeningitis interna haemorrhagica was present in 9 cases and 5 of these have been fully investigated by Russell and Cairns (1934) who conclude that the haemorrhage is consequent upon dilatation and rupture of the capillaries of the areolar layer of the dura, following permeation by growth of the veins and capillaries of the outer layer of the dura from the adjacent skull.

The sites of the primary growths in the cases showing diffuse involvement of the leptomeninges are shown in Table VIII. In these 6 cases there was obvious naked-eye evidence of growth in the leptomeninges ; to a considerable extent the ability to recognize this infiltration depends upon the experience of the morbid anatomist.

TABLE VIII.—*Site of Primary growth in Cases with Diffuse Involvement of the Leptomeninges.*

| Sites of primary growths. | Number. |
|---|---|
| Stomach . . . . | 2 |
| Testis . . . . | 1 |
| Lung . . . . | 3 |
| Total . . . . | 6 |

*The mode of spread of growth to the leptomeninges.*

The suggestion that growth can reach the brain by retrograde lymphatic embolism from affected cervcial lymph glands has been dismissed by Willis (1934), on the grounds that no lymphatics exist in the central nervous system or its meninges. The 2 cases in this series in which meningitis carcinomatosa followed primary carcinoma of the stomach showed no macroscopic evidence of growth in the cervical tissues.

Carcinomatous meningitis is usually found to be associated with, and evidently referable to the breakdown of a discrete secondary deposit in the brain into the ependyma lining the ventricular system. If it arose from secondary nodules in the substance of the brain encroaching on the surface and then spreading diffusely, it should be more frequently encountered. In this series many cases were found in which nodules had actually broken through the leptomeninges but there was no evidence of a generalised cancerous meningitis. In occasional cases we have failed to demonstrate a ventricular deposit ; in these it may have been missed or, alternatively, direct embolism to the meninges may have occurred by the blood stream. It is noteworthy that this ventricular deposit may be small, and clinically silent, the neurological symptoms being referable to the meningeal spread and the involvement of nerve-roots, especially in the posterior fossa.

Invasion of the leptomeninges by secondary growth is frequently followed by perivascular infiltration of the cortex and, except for the different morphology of the cells, resembles closely the perivascular cuffing found in encephalitis lethargica. On the other hand, discrete nodules of secondary growth in the substance of the brain very rarely show this perivascular infiltration at their periphery.

### *Involvement of the Pituitary Gland by Secondary Growth.*

The sites of the primary growths in 14 cases in which there was involvement of the pituitary are shown in Table IX.

TABLE IX.—*Site of the Primary Growth in Cases with Involvement of Pituitary.*

| Sites of primary growths. | Number of cases. |
|---|---|
| Nasopharynx . . . | 4 |
| Lung . . . . | 5 |
| Prostate . . . . | 1 |
| Breast . . | |
| Stomach . . . . | 1 |
| Middle ear . . . . | 1 |
| Not determined . . . | 1 |
| Total . . . . | 14 |

*Invasion of the Spinal Cord and its Meninges by Secondary Growth.*

It is well recognized that the vertebral column may be invaded by secondary growth producing evidence of compression of the cord without actual transgression of the theca. In this series only those cases in which the spinal cord or its meninges were invaded by secondary growth are considered. Ten such cases were found, and the sites of the primary tumours are shown in Table X.

TABLE X.—*Site of Primary Growth in Cases with Involvement of the Spinal Cord.*

| Sites of primary growths. | Number. |
|---|---|
| Lung . . . . | 6 |
| Stomach . . . . | 1 |
| Testis . . . . | 1 |
| Breast . . . . | 1 |
| Sarcoma of rib . . . | 1 |
| Total . . . . | 10 |

In 2 cases primary tumours, arising in the stomach and a rib respectively, produced metastases in the epidural tissues with direct infiltration of the meninges of the cord from the outer surface.

In 2 cases there were isolated nodules of growth in the cord substance as well as cerebral metastases. Necropsy in the first revealed an unsuspected primary carcinoma of the lung with a metastasis in the cerebellum and another secondary nodule occupying the first and second lumbar segments of the cord. In the second case multiple secondary deposits involved the brain while a secondary intramedullary nodule occupied the fifth lumbar and first sacral segments of the cord. In this case a radical mastectomy for carcinoma had been performed 2 years previous to necropsy.

The remaining 6 cases showed infiltration of the spinal leptomeninges, and in all except one there was involvement of the meninges or substance of the brain, or both.

*Discussion of the Non-Pulmonary Group.*

The non-pulmonary cases form a heterogenous group and each category includes generally only a few cases. Metastatic growths from the breast and alimentary canal together account for 40 out of the total of 99 cases.

The effect of selection, which has been previously discussed, is apparent when the 20 examples arising from primary carcinoma of the breast are considered. Willis (1948, p. 244) states that cerebral metastases are present in about 20 per cent of fatal cases of mammary carcinoma and that in fact this is the most frequent source of such metastases. Figures from published series are quoted in his second edition (Willis, 1952). Thus, if the effect of selection were eliminated, there should be a closer approximation between the total figures for the pulmonary and mammary groups.

Consideration of the alimentary canal as a source of cerebral metastases shows that the group of 20 cases includes 13 examples arising from gastric carcinoma. The effect of selection on this group is much less pronounced, and the low figure indicates the greatly reduced tendency for cerebral metastases to occur in cases of gastric carcinoma, when compared with pulmonary carcinoma.

The very great tendency of chorion carcinoma to metastasize to the brain has been noted by Willis (1952, p. 98), and the low figure in the present series reflects only the great rarity of this condition.

This heterogeneous group includes many other cases of great pathological interest, some of which have been briefly mentioned, but a more detailed consideration of these is not germane to the present communication.

### SUMMARY AND CONCLUSIONS.

A series of 216 necropsies, in which the central nervous system and meninges were involved by secondary growth, has been analyzed in terms of the primary site of the neoplasm. While a great majority (117 cases) was attributed to primary carcinoma of the bronchus, it has been argued that this figure is inflated by reason of selective factors. These have also depressed the total due to mammary carcinoma (20 cases).

Analysis of the pulmonary group shows that a single cerebral metastasis was identified in 30 per cent of cases, but in 2 only of these was this the sole metastasis in the body. No significant difference was found in the frequency of involvement of the right and left cerebrum, but there was a predilection for the cerebellum which remains unexplained. On histological examination the oat-cell type of bronchial carcinoma was predominant, in conformity with its incidence in these primary growths as a whole.

We wish to thank Professor D. S. Russell for her help and advice in the preparation of this paper.

### REFERENCES.

ADLER, I.—(1912) 'Primary Malignant Growths of the Lungs and Bronchi.' New York (Longmans, Green & Co.), 1st ed.
BARRON, M.—(1922) *Arch. Surg., Chicago*, **4**, 624.
BOYD, W.—(1931) 'Pathology of Internal Disease.' London (H. Kimpton), 1st ed., p. 830.
BRAIN, W. R.—(1933) 'Diseases of the Nervous System.' London (Oxford University Press), 1st ed., p. 204.
BRIESE.—(1920) *Frankfurt. Z. Path.*, **23**, 48.
BROUWER, B.—(1932) *Psychiat. neurol. Bl., Amst.*, **36**, 108.
CAIRNS, H.—(1926) *Lancet*, i, 845.
COURVILLE, C. B.—(1950) 'Pathology of the Central Nervous System.' Mountain View, California (Pacific Press Publishing Association), 3rd ed., p. 426.
CUSHING, H.—(1932) 'Intracranial Tumours.' Springfield, Illinois (C. C. Thomas), 1st ed., p. 105.
DOSQUET,—(1921) *Virchows Arch.*, **234**, 481.
DUNLAP, H. F.—(1930) *Proc. Mayo Clin.*, **5**, 372.—(1932) *Ann. intern. Med.*, **5**, 1274.
ELKINGTON, J. ST. C.—(1935) *Proc. R. Soc. Med.*, **28**, 1080.
FERGUSON, F. R., AND REES, W. E.—(1930) *Lancet*, i, 738.
FLAVELL, G.—(1949) *Brit. med. J.*, **2**, 736.
FRIED, B. M., AND BUCKLEY, R. C.—(1930) *Arch. Path. Lab. Med.*, **9**, 483.
GALLAVARDIN, L., AND VARAY, F.—(1903) *Rev. Médecine*, **23**, 441, 561.
GLOBUS, J. H., AND MELTZER, T.—(1942) *Arch. Neurol. Psychiat., Chicago*, **48**, 163.
GRANT, F. C.—(1926) *Ann. Surg.*, **84**, 635.

HENSON, R. A., CRAWFORD, J. V., AND CAVANAGH, J. B.—(1953) *J. Neurol. Psychiat.*, **16**, 127.

KRASTING, K.—(1906) *Z. Krebsforsch.*, **4**, 315.

MANGANIELLO, L. O. J., REIMANN, D. L., AND WAGNER, J. A.—(1948) *Arch. Neurol. Psychiat., Chicago*, **59**, 99.

MEAGHER, R., AND EISENHARDT, L.—(1931) *Ann. Surg.*, **93**, 132.

PARKER, H. L.—(1927) *Arch. Neurol. Psychiat., Chicago*, **17**, 198.

RUSSELL, D. S., AND CAIRNS, H.—(1934) *Brain*, **57**, 32.

SEYFARTH, C.—(1924) *Dtsch. med. Wschr.* **50**, 1497.

SIMPSON, S. L.—(1929) *Quart. J. Med.*, **22**, 413.

TURNBULL, H. M.—(1911) *Trans. med. Soc., Lond.*, **34**, 240.

WILLIS, R. A.—(1934) 'The Spread of Tumours in the Human Body.' London (Churchill), 1st ed., p. 351.—(1948) 'Pathology of Tumours.' London (Butterworth & Co.), 1st ed.—(1952) 'The Spread of Tumours in the Human Body.' London (Butterworth & Co.), 2nd ed.